# In Vitro and In Vivo Comparison Study of Electrospun PLA and PLA/PVA/SA Fiber Membranes for Wound Healing

**DOI:** 10.3390/polym12040839

**Published:** 2020-04-06

**Authors:** Hongyan Bi, Tianyi Feng, Binbin Li, Yingchao Han

**Affiliations:** State Key Laboratory of Advanced Technology for Materials Synthesis and Processing, Biomedical Materials and Engineering Research Center of Hubei Province, Wuhan University of Technology, Wuhan 430070, China; y13277937880@163.com (H.B.); 13890253939@163.com (T.F.)

**Keywords:** tissue repair, wound healing, wound dressing, fiber membrane, electrospinning

## Abstract

Wound dressings can accelerate wound healing. The degradable polymer poly(lactic acid) (PLA) shows good mechanical properties and biocompatibility. Sodium alginate (SA) holds good biocompatibility, hemostasis, and high hygroscopicity. Poly(vinyl alcohol) (PVA) has good spinnability as a pharmaceutical excipient. Herein, we carried out a comparison study of electrospun PLA and PLA/PVA/SA fiber membranes for wound healing in vitro and in vivo. In this study, PLA and PLA/PVA/SA nanofiber membranes were fabricated through electrospinning to produce a highly porous and large specific surface area that could promote wound healing. In vitro experiments showed that PLA and PLA/PVA/SA nanofiber membranes could all provide good support for the growth of rat fibroblasts (L929). Moreover, rat fibroblasts displayed slightly better adhesion and proliferation on PLA/PVA/SA than on the PLA fiber membranes. The in vivo potentiality of the PLA and PLA/PVA/SA fiber membranes was assessed in rat models of skin defects in which the PLA and PLA/PVA/SA fiber membranes significantly improved wound healing compared to commercially available gauzes. No significant differences in wound healing were observed between PLA and PLA/PVA/SA fiber membranes in our study. Furthermore, Masson staining and PCR displayed the PLA fiber membrane promoted protein deposition compared to the PLA/PVA/SA fiber membrane. In addition, IHC suggested that PLA/PVA/SA dressing reduced the inflammatory response during early wound healing compared to the PLA fiber membrane. These findings highlight the utility of fiber membranes as novel wound-healing dressings.

## 1. Introduction

Tissue repair is a complex biological process that involves an array of biological pathways [1]. Wound dressings have been used since the dawn of time. Traditional dressings such as gauze and bandages directly contact the wound, which can become attached to some of the dressing’s fibers. Removing the dressing would lead to pain and reopening, delaying wound healing. An ideal dressing should improve wound healing, prevent infection, and provide a wet and isolated environment for wound closure. In addition, the dressing should be degradable or replaceable without adverse effects or pain [2]. Sponges, hydrogels, and fibers [3,4,5,6,7] have emerged as promising wound dressings materials, of which fibers have attracted intense research attention [8,9].

To date, various methods have been developed to produce fibers, including electrospinning, which uses electrostatic forces to produce fibrous polymer structures of differing diameters. Electrospinning is a simple and effective method, adjusting and controlling the diameter, shape, and surface features of the fibers [10,11]. The fiber membrane fabricated by electrospinning technology displays good porosity and pore interconnectivity, and a large, specific surface area for cell adherence [12,13]. The advantage of electrospun fibers is their ability to mimic the extracellular matrix (ECM), thereby preventing scar formation by improving hemostasis and the absorption of wound exudate [3,11]. However, although electrospinning is now rapidly developing the coaxial [14], triaxial [15], side-by-side [16], and multifluid [17] processes for creating core-shell, trilayer core-shell, Janus, and other complex nanostructures, their applications are almost at the stage of concept demonstration [18]. How to deepen the real applications of electrospun fibers, even the commercial products, poses a big challenge to the researchers.

In recent years, a number of polymers have been used to fabricate electrospun wound dressings, including collagen, chitosan, poly(vinyl alcohol) (PVA), and poly(caprolactone) [5,7,19]. Of particular interest is poly(lactic acid) (PLA), due to its cost-effective production from sugar beets and corn [20]. PLA displays high biocompatibility, degradability, mechanical properties, and strength [21], and has been used in an array of biomedical field applications, including degradable sutures, drug delivery materials, nanoparticles, and porous scaffolds [21,22,23]. Sodium alginate (SA) is a natural polymeric material. Given its high cytocompatibility, biodegradability, and antibacterial characteristic, SA has wide applications in the biomedical field, such as tissue engineering, biomedicine, and drug delivery systems [24]. When used as a wound dressing, it absorbs wound exudate and maintains a moist microenvironment [25]. Moreover, SA holds hemostatic properties, which shorten the inflammatory phase of wound recovery and accelerates skin wound healing [26]. However, the rigidness and large brittleness limit its application [27]. With the aid of PVA, SA electrospun fiber membrane can be achieved in an aqueous system [7]. PVA is a synthetic but biocompatible polymer, and it is also being used widely for various medical applications as a medicinal excipient. It has been reported that in vitro synthetic electrospun PLA scaffolds can, as a dermal substitute, induce skin cell migration and proliferation along the fibers of the scaffold to form new collagen [28]. Nguyen et al. produced curcumin-loaded PLA fibers and assessed their in vivo wound healing capability in rat models, displaying effective wound-healing performance [29]. Kai Chen et al. constructed a silver-loaded PVA/SA/CMCS hydrogel antibacterial wound dressing, which exhibited good mechanical properties, antibacterial activity, and biocompatibility [30]. In one study, the honey-loaded alginate/PVA nanofibrous membrane was fabricated using electrostatic spinning technology. The nanofibrous membranes with increasing honey content showed enhanced antioxidant activity, suggesting the ability of nanofibrous dressings to control the overproduction of reactive oxygen species [31]. In this study, PLA and PLA/PVA/SA fibers were fabricated through electrospinning, and their abilities to enhance tissue repair in wound models were compared to reveal the effect of the component. Fibers bioactivity was investigated through cytotoxicity, cell adhesion, and proliferation by fibroblast cells (L929). Using full-thickness skin defect rat models, healing efficiency and scar formation were assessed in models treated with PLA and PLA/PVA/SA fiber membranes. We further assessed the inflammatory response, collagen deposition, and new blood vessel regeneration at the molecular level.

## 2. Methods

### 2.1. Materials

*N*,*N*’–dimethylformamide (DMF) and dichloromethane were obtained from Sinopharm. PLA, with an average molecular weight of 1,000,000 (DG-DL400), was purchased from Jinan Daigang Biomaterials. PVA and SA were purchased from Shanghai Aladdin Biochemical Technology Co., Ltd. (Shanghai, China)

### 2.2. Preparation and Characterization of the Materials

PLA fiber and PLA/PVA/SA composite fiber were prepared using the electrospinning method. First, PVA and SA were dissolved in purified water to get the PVA/SA spinning solution. PLA was dissolved in dichloromethane/DMF (2/1, *v/v*) to get the PLA spinning solution. The ambient temperature was 25 °C, and the relative humidity was 40–50% in a closed chamber. Scanning electron microscopy (SEM, JSM-IT200) was employed to confirm fiber production.

For the preparation of PLA fiber, the PLA spinning solution was placed in 10 mL syringe having a needle with an internal diameter of 510 μm. An aluminum foil was used as a collector with a distance of 20 cm from the tip of the needle. The voltages of the needle tip and aluminum foil were set at +8 kV and −2.5 kV, respectively. The electrospinning was carried out at a flow rate of 0.01 mm/min. We explored the effects of PLA concentration on the morphology and diameter of composite fibers.

For the preparation of PLA/PVA/SA composite fiber, PVA/SA fibers were collected on the above PLA fiber with aluminum foil. The voltages of the needle tip and aluminum foil were set at +11 kV and −2.5 kV, respectively. The electrospinning was carried out at a flow rate of 0.1 mm/min. We explored the effects of SA contents on the morphology and diameter of composite fibers. Samples were characterized by using the Fourier transform infrared spectroscopy (FT-IR, Thermo Nicolet 6700, Waltham, MA, USA) and contact-angle measuring instrument (JC200C, Shanghai zhongchen digital technic apparatus co.,ltd, China). 

### 2.3. In Vitro Cytocompatibility

PLA and PLA/PVA/SA fibers were UV-sterilized for 12 h and seeded into culture plates with L929 cells (Shanghai Cell Bank, Chinese Academy of Sciences, Shanghai, China) in RPMI medium at 37 °C in 5% CO_2_ for 1, 3, and 7 d. For fluorescence microscopy, cells were fixed in 4% PFA (Google Biotechnology, Wuhan, China) and DAPI (Google Biotechnology) -stained. Cells were counted and imaged using a microscope (OLYMPUS IX51, Tokyo, Japan) and analyzed by the MicroPublisher Q-IMAGING system (Surrey, Canada).

For SEM (TESCAN VEGA 3 LMU, Brno, Czech Republic), cells were immobilized, dehydrated, and dried prior to imaging. 

### 2.4. Animal Experiments

Thirty-six Sprague Dawley (SD) male rats weighing about 200 g were divided into three groups. The SD male rats (~200 g) were anesthetized with pentobarbitone sodium, and their back hair was shaved. The skin area was disinfected with alcohol, and a wound (1.5 cm by 1.5 cm and full-thickness) was produced on the back of each rat [5]. Wounds were PLA-dressed, PLA/PVA/SA-dressed, or treated with commercial gauzes as controls. Animals were caged individually and provided free access to food and water. Changes in weight were recorded, and wound healing was assessed at defined times from 1–16 days. The wound size was calculated using the following equation: wound healing rate (%) = (*S*_0_ − *S_t_* )/*S*_0_ × 100%, where *S*_0_ is the initial wound area (*t* = 0) and *S_t_* is the wound area at a time interval ‘‘*t* > 1”. Animals were sacrificed at the specified time points.

### 2.5. Histology

Wound tissue was harvested from days 7–21 and fixed in 4% PFA. Tissues were sectioned (4 μm) for H and E and Masson’s trichrome staining. Sections were imaged in a light microscope (Nikon Eclipse CI).

### 2.6. Immunohistochemistry

Sections were paraffin-embedded, dehydrated, and probed with antibodies against Collagen I and TNFα (Google Biotechnology) at 4 °C for 8 h. Sections were washed in PBS and labeled with the indicated secondary antibodies for 4 h at 4 °C in the dark. Sections were imaged on an optical microscope (Nikon Eclipse CI).

### 2.7. Western Blot Analysis

Tissues and cells were lysed in a RIPA buffer supplemented with protease inhibitors (Google Biotechnology). Lysates were centrifuged at 15,000 rpm for 5 min and resolved by SDS-PAGE. Proteins were then transferred to PVDF membranes (PVH00010, Millipore, Burlington, MA, USA) and probed overnight with rat anti-CD31 or rat anti-Collagen I antibodies (Google Biotechnology, China). Membranes were then washed and labeled with HRP conjugated secondary antibodies at room temperature. Band intensities were analyzed using Alpha software (AlphaEaseFC, Alpha Innotech, San Leandro, CA, USA).

### 2.8. qRT-PCR Analysis

The mRNA levels of CD31 and Collagen I were normalized to GAPDH. Tissues were lysed and total RNA was extracted. qPCR was performed using FastStart Universal SYBR Green Master (Rox). PCR parameters: 95 °C for 10 min; 40 cycles at 95 °C for 15 s and 60 °C for 60 s. PCRs were performed on an Applied Biosystems 7500 Real-Time PCR System and relative expression quantified via the 2^−ΔΔCT^ method. Melting curves were produced to confirm successful amplification. At least three samples per test were taken for statistical analysis.

## 3. Results and Discussion

### 3.1. Morphology and Performance of Material

**Figure 1:**
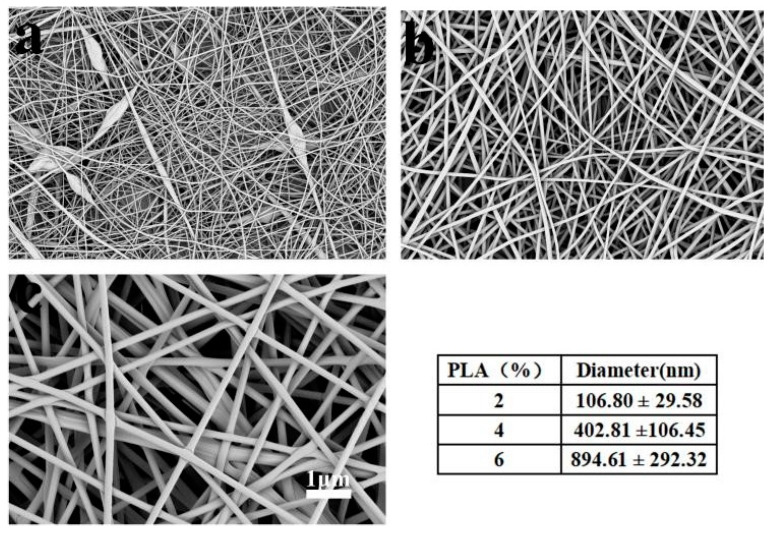
The morphologies and diameters of the poly(lactic acid) (PLA)fiber membranes, (**a**) 2%; (**b**) 4%; and (**c**) 6%.

Figure 1 shows the morphology of the PLA fiber membranes. It can be seen that the sample had beaded fibers while the PLA concentration was at 2%. With 4% of PLA concentration, the fibers showed good morphology and uniform diameter (402.8 ± 106.6 nm). With the increase of PLA concentration to 6%, the diameter of fibers became larger (894.6 ± 292.3 nm). So, PLA (4%) solution was used to prepare the PLA fiber membrane.

**Figure 2:**
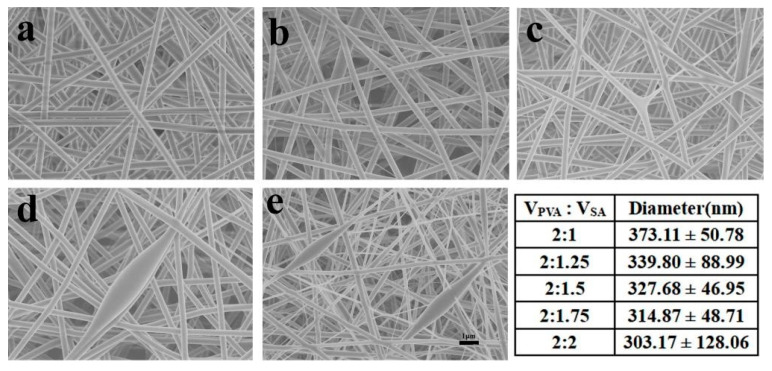
The morphologies and diameters of the poly(vinyl alcohol) (PVA)/sodium alginate (SA) fiber membranes, V_PVA_:V_SA_ (**a**) 2:1; (**b**) 2:1.25; (**c**) 2:1.5; (**d**) 2:1.75; and (**e**) 2:2.

**Figure 3:**
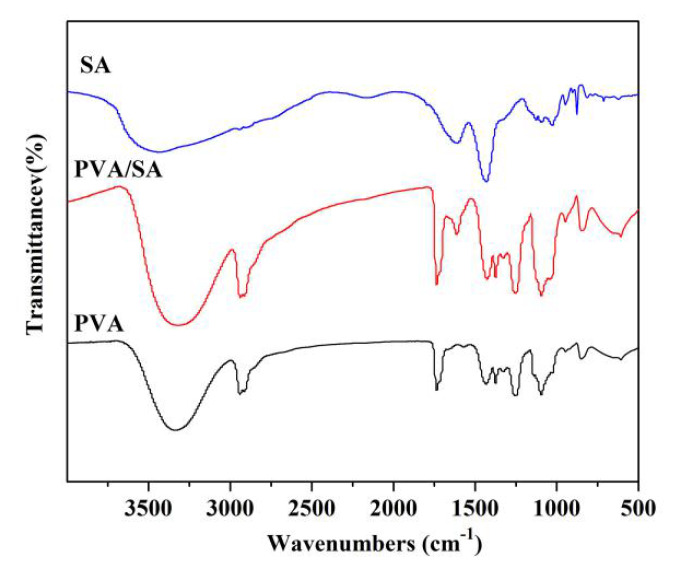
FTIR spectra of samples.

**Figure 4:**
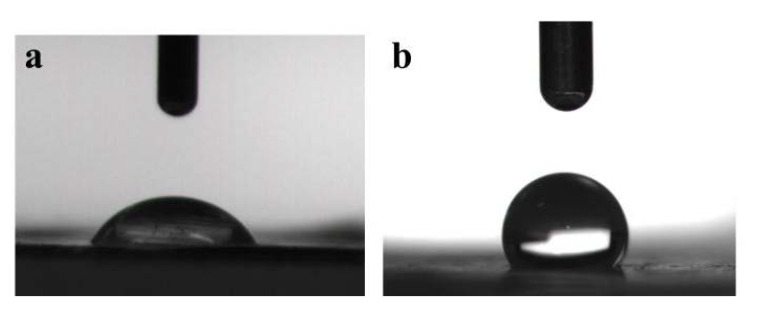
The contact angles of the PLA/PVA/SA composite fiber membrane, (**a**) the top layer of the PVA/SA fiber membrane, and (**b**) the bottom layer of the PLA fiber membrane.

Figure 2 shows the morphologies and diameters of PVA/SA fibers in PLA/PVA/SA composite membranes. It can be seen that PVA/SA fibers had good morphology and uniform diameter, while the volume ratio of PVA to SA was changed from 2:1 to 2:1.5. However, while the volume ratio of PVA to SA was changed from 2:1.75 to 2:2, the beaded fibers appeared. The diameters of PVA/SA fibers decreased with the increase of SA content. This was mainly because SA was a polyelectrolyte, and the addition of SA would adversely affect the spinning performance of PVA. So, PVA/SA(V_PVA_:V_SA_ = 2:1) solution was used to prepare the PLA/PVA/SA composite membrane. The FTIR spectra of the sample are shown in Figure 3. There were vibrational peaks at 1609 cm^−1^ (-COO- group), 1035 cm^−1^ (-COC- group), and 947 cm^−1^ (O-H) in PVA/SA fiber membranes, indicating that SA has been successfully added to the composite fiber membrane. The contact angle of the PLA/PVA/SA composite fiber membrane was shown in Figure 4. The contact angle of the top layer of the PVA/SA fiber membrane was 59.5°, and the contact angle of the bottom layer of the PLA fiber membrane was 129°, showing the hydrophilic top layer and hydrophobic bottom layer of the PLA/PVA/SA composite fiber membrane. 

Both fiber membranes possess porous structures as the fibers stack on top of each other, resembling that of the native ECM. This morphology benefits cell adhesion and provides a platform for efficient cell proliferation. The structure also benefits fluid absorption, hemostasis, and gaseous exchange, producing a humid environment that favors wound healing and tissue regeneration.

### 3.2. In Vitro Compatibility

**Figure 5:**
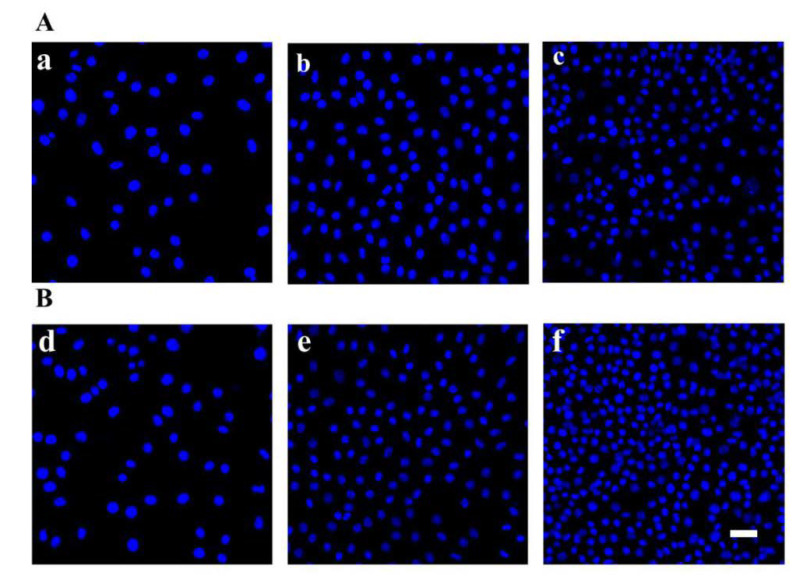
(**A**) Fluorescence microscopy of L929 cells adhered to the PLA membranes on days (**a**) 1, (**b**) 3, and (**c**) 7. (**B**) Fluorescence microscopy of L929 cells adhered to the PLA/PVA/SA membranes on days (**d**) 1, (**e**) 3, and (**f**) 7.

**Figure 6:**
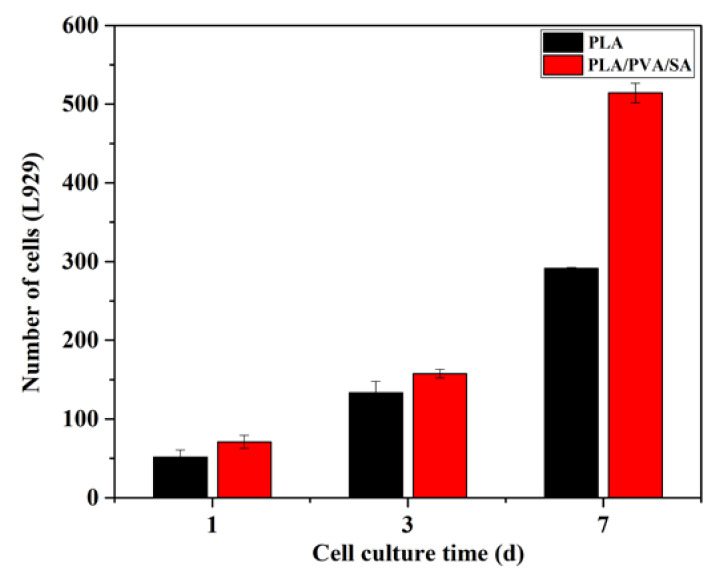
L929 cell counts on PLA and PLA/PVA/SA membranes at the indicated cultured periods.

**Figure 7:**
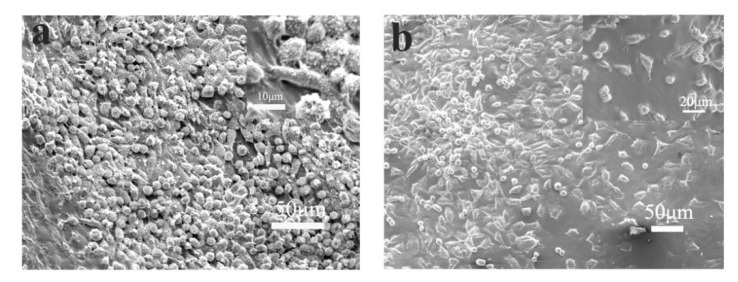
SEM image of L929 cells on day 7 of (**a**) PLA fibers; (**b**) PLA/PVA/SA fibers.

The adhesion of L929 cells cocultured on different scaffolds was evaluated by nuclear staining. As shown in Figure 5, L929 cells could adhere and proliferate on PLA and PLA/PVA/SA fiber membranes well. Cell counts (Figure 6) showed that the numbers of L929 cells seeded on PLA and PLA/PVA/SA fiber membranes increased with culture time, respectively. Moreover, there was no obvious difference in cell number between PLA and PLA/PVA/SA fiber membranes, while culture time was 1 d and 3 d. However, with the increase of culture time to 7 d, the cell number on PLA/PVA/SA fiber membrane was obviously higher than that of PLA fiber membrane. SEM observations (Figure 7) further displayed the morphology of L929 cells seeded on PLA and PLA/PVA/SA fiber membranes. Compared to the cells on the PLA fiber membrane, the cells on the PLA/PVA/SA fiber membrane tended to spread out muh more and embed into the membrane. These indicated that the addition of PVA/SA fibers seemed to be helpful for the adhesion and proliferation of cells due to their good hydrophilicity.

### 3.3. In Vivo Assessments

**Figure 8:**
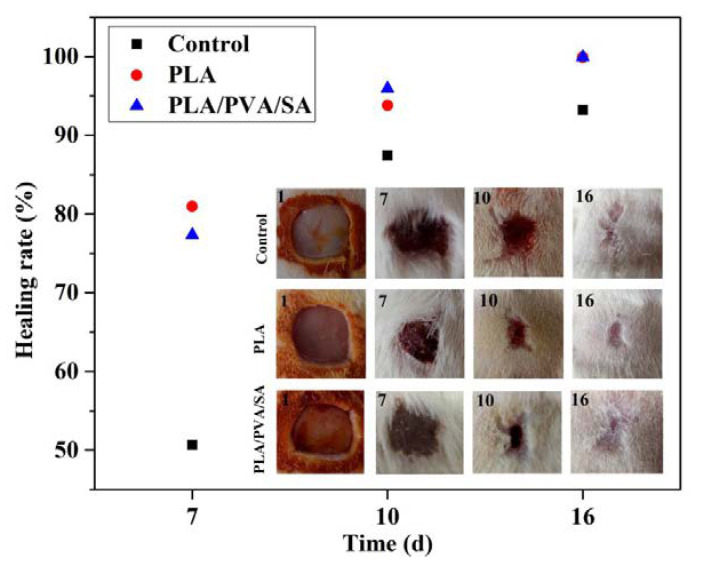
Healing rate and wound closure images in PLA fiber-treated and PLA/PVA/SA fiber-treated on days 1, 7, 10, and 16.

The effects of the dressings on in vivo wound healing were analyzed using Image J software on days 7, 10, and 16 postsurgery. Figure 8 showed that the healing of dressing-treated wounds was faster than that of the control group in general, and there was no obvious difference in the healing of wounds between PLA and PLA/PVA/SA groups. At day 7, PLA and PLA/PVA/SA dressing-treated wounds contracted 80.96% and 77.33%, which were much higher than that of commercial gauzes (50.67%). On day 10, wounds in the dressings group had a larger contraction of about 95% than that of commercial gauzes group (87.42%). On day 16, the wound had almost completely healed in the PLA and PLA/PVA/SA groups, with minimal scarring evident. These data suggested that the biocompatibility and porous nature of PLA materials benefitted wound healing, maintaining good fiber morphology as a wound dressing. SA is biocompatible and highly hygroscopic, which is able to absorb wound exudate, maintaining a moist microenvironment to shorten the inflammatory phase of wound recovery and accelerate skin wound healing. However, SA absorbs interstitial fluid, resulting in the damage of the microstructure of the dressing. In addition, the high surface-area-to-volume ratio and porous nature of the fiber mats promoted an ideal environment for the absorption of exudate, gas, and fluid exchange, and protection from bacterial infection [29]. The biodegradable fibrous scaffolds, therefore, promoted wound repair. From a tissue-engineering standpoint, the fibers had high cell conductivity and could guide tissue regeneration, all of which facilitates wound healing and skin regeneration [9].

### 3.4. Histological Assessments

**Figure 9:**
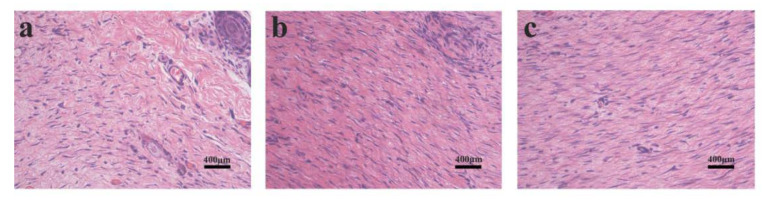
H and E staining images of the wound tissue on day 16. (**a**) Control; (**b**) PLA-treated; and (**c**) PLA/PVA/SA-treated.

To observe the effects of PLA and PLA/PVA/SA dressings on wound healing, we examined the histological changes in the skin of SD rats by H and E staining (Figure 9). As shown in Figure 9a, the wound area displayed significant inflammation with loosely bound and disordered collagen fibers in the control group, where wounds showed poorer healing. Compared to the control group, the wound areas of dressings in treated groups (Figure 9b,c) were covered with denser and ordered collagen fibers. The two dressing-treated groups showed similar results. Only the collagen fibers of the PLA group seemed to be a little denser than that of the PLA/PVA/SA group. Histology analysis revealed that the PLA and PLA/PVA/SA dressings could enhance the regeneration of collagen fibers for accelerating wound healing.

### 3.5. Collagen Deposition

**Figure 10:**
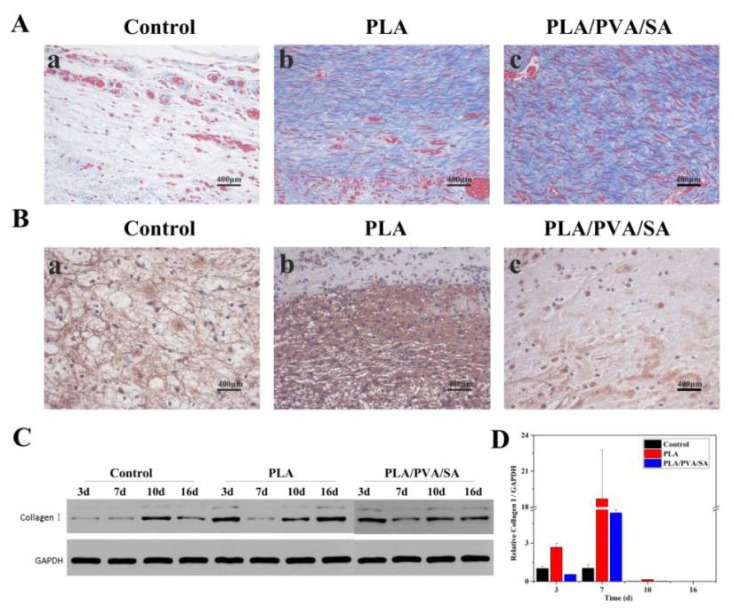
Effects of PLA dressings on wound collagen deposition. (**A**) Masson’s staining on day 16. (**B**) Immunohistochemistry analysis of Collagen I (day 3) (**a**) control; (**b**) PLA group; (**c**) PLA/PVA/SA group. (**C**) Western blot of Collagen I, (**D**) quantitative analysis of the Collagen I deposition.

Collagen is an integral component of healthy skin and is present in large doses in areas of tissue regeneration and cell proliferation [27]. Masson’s staining results showed that on day 16, the collagens were intermittently found in the control group, indicating incomplete wound healing (Figure 10A(a)), and the wound areas of dressing-treated groups (Figure 10A(b,c)) showed relatively denser and more continuous collagen (blue staining). The two groups treated by dressings showed similar results. Only the collagen fibers of the PLA group seemed to be denser than that of the PLA/PVA/SA group. IHC (Figure 10B) showed that the expression of Collagen I in the PLA group was significantly higher on day 3 compared to other groups. The Western blotting results showed that Type I Collagen in the PLA-treated group exhibited higher expression levels than that of other groups, especially when compared to the control group on days 3 and 16 (Figure 10C). Moreover, collagen expression was also enhanced in the PLA dressings, especially on day 7, but steadily declined from days 7 to 10 when the majority of the wound had been repaired (Figure 10D). The results of WB are not completely consistent with the results of PCR. This may be because PCR detects the target gene at the transcription level. There is a complicated process from transcription to translation, so the expression level of the gene and the expression level of the protein are not necessarily positively correlated. From most of the results, PLA and PLA/PVA/SA can promote protein deposition and accelerate the wound healing process. PLA fibers can still maintain fiber shape when exposed to interstitial fluid. 

### 3.6. Effects of PLA and PLA/PVA/SA Fiber Membranes on Wound Angiogenesis

**Figure 11:**
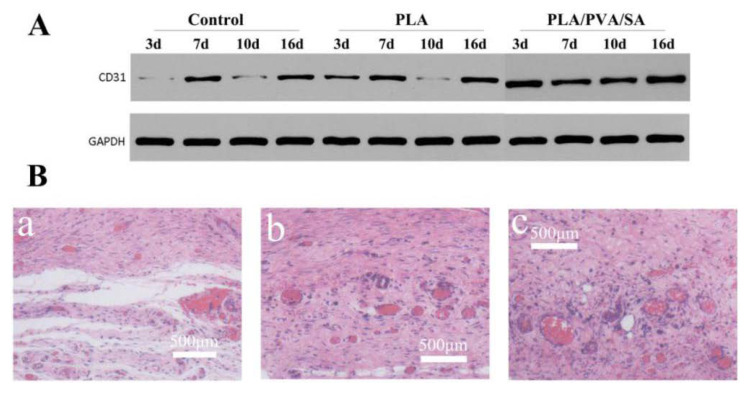
(**A**) Western blot of CD31. (**B**) Images of vessel formation of the wound at 16 days; (**a**) control; (**b**) PLA group; (**c**) PLA/PVA/SA group.

CD31 is expressed during the early stages of vascular development during wound healing [32]. Western blot (Figure 11A) displayed the CD31 expression level of the PLA/PVA/SA-treated group as having a significantly higher density than that of the PLA and control group during the healing process. As shown in Figure 11B, there were few blood vessels in the wound area of the control group. The area and number of blood vessels around the wound were significantly larger in PLA and PLA/PVA/SA than that of the control group on day 16 (Figure 11B). Meanwhile, the number of blood vessels in PLA was not significantly different from that in PLA/PVA/SA group. The blood vessels in PLA/PVA/SA seemed to be thicker than those in the PLA group. These results suggested that both PLA and PLA/PVA/SA dressings significantly enhance angiogenesis, highlighting their promise in wound healing.

### 3.7. PLA and PLA/PVA/SA Membranes Prevent Inflammation

**Figure 12:**
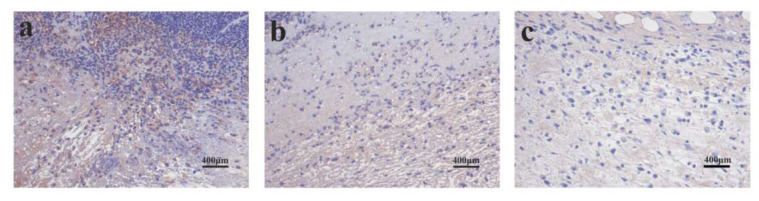
Immunohistochemistry of TNF-α (day 3) in (**a**) control, (**b**) PLA, and (**c**) PLA/PVA/SA groups.

The effects of the dressings on the inflammatory response were assessed in vivo by analyzing the expression of TNF-α (Figure 12). Figure 12a showed that the expression of TNF-α in the control group was very obvious on day 3, indicating a severe inflammatory response. Figure 12b showed that a certain expression of TNF-α in the PLA group indicates the presence of an inflammatory response on day 3. IHC revealed that TNF-α levels in the PLA/PVA/SA groups were significantly lower than the control and PLA group on day 3, suggesting that PLA/PVA/SA dressings reduce inflammatory responses during early wound healing. This may be due to the anti-inflammatory and antibacterial activities of SA [27].

SA is biocompatible and highly hygroscopic, and is able to absorb wound exudate, maintaining a moist microenvironment to shorten the inflammatory phase of wound recovery and accelerate skin wound healing. Meanwhile, SA absorbs interstitial fluid, which damages the microstructure of the fibers. The biocompatibility and porous nature of PLA materials benefitted wound healing. The good mechanical properties of the PLA are conducive to the fit of the dressings on the wound surface. Due to their random alignment and diameter, fiber membranes prepared by electrospinning mimic the ECM. The natural ECM is a noncellular component present in all tissues that regulate wound healing through the physical support of cells, promoting their division, differentiation, and migration [33,34,35]. Nanofibrous meshes promote hemostasis of injured tissues due to their small interstices and their high surface area [36]. Nanofiber membranes have a highly-interconnected porous structure [37] that permits high levels of cell respiration and gas permeation, preventing desiccation and dehydration to promote wound healing.

## 4. Conclusions

PLA and PLA/SA/PVA fiber membranes were fabricated by electrospinning, and exhibited promising biocompatibility. L929 cells could better adhere and proliferate on PLA/PVA/SA fibers. In vivo wound healing experiments and histological examinations showed that the PLA and PLA/SA/PVA fiber membranes could accelerate the rate of wound closure compared to commercial gauzes. In vivo assessments showed that both PLA and PLA/PVA/SA fiber membranes had positive effects on collagen deposition, angiogenesis, and inflammation, compared to the control group. PLA fiber membrane showed higher collagen deposition than PLA/PVA/SA fiber membrane. Both fiber membranes displayed similar angiogenesis, and merely the blood vessels in the PLA/PVA/SA group seemed to be thicker than that of the PLA group. In addition, PLA/PVA/SA dressings significantly reduced inflammatory responses during early wound healing.
